# Overexpression of* AaWRKY1* Leads to an Enhanced Content of Artemisinin in* Artemisia annua*

**DOI:** 10.1155/2016/7314971

**Published:** 2016-03-28

**Authors:** Weimin Jiang, Xueqing Fu, Qifang Pan, Yueli Tang, Qian Shen, Zongyou Lv, Tingxiang Yan, Pu Shi, Ling Li, Lida Zhang, Guofeng Wang, Xiaofen Sun, Kexuan Tang

**Affiliations:** Key Laboratory of Urban Agriculture (South) Ministry of Agriculture, Plant Biotechnology Research Center, Fudan-SJTU-Nottingham Plant Biotechnology R&D Center, School of Agriculture and Biology, Shanghai Jiaotong University, Shanghai, 200240, China

## Abstract

Artemisinin is an effective component of drugs against malaria. The regulation of artemisinin biosynthesis is at the forefront of artemisinin research. Previous studies showed that AaWRKY1 can regulate the expression of* ADS*, which is the first key enzyme in artemisinin biosynthetic pathway. In this study,* AaWRKY1* was cloned, and it activated ADSpro and CYPpro in tobacco using dual-LUC assay. To further study the function of AaWRKY1, pCAMBIA2300-AaWRKY1 construct under 35S promoter was generated. Transgenic plants containing* AaWRKY1* were obtained, and four independent lines with high expression of* AaWRKY1* were analyzed. The expression of* ADS* and* CYP*, the key enzymes in artemisinin biosynthetic pathway, was dramatically increased in* AaWRKY1-*overexpressing* A. annua* plants. Furthermore, the artemisinin yield increased significantly in* AaWRKY1-*overexpressing* A. annua* plants. These results showed that AaWRKY1 increased the content of artemisinin by regulating the expression of both* ADS* and* CYP*. It provides a new insight into the mechanism of regulation on artemisinin biosynthesis via transcription factors in the future.

## 1. Introduction

*A. annua* is an important traditional Chinese herbal plant according to the Chinese materia medica [1]. Artemisinin, extracted from herbal plants* A. annua*, is an effective component of drugs against malaria. It was discovered by Miller and Su [2]. Artemisinin-based combination therapy (ACT) is considered the most effective method against malaria [3]. In recent years, other functions of artemisinin have been reported. For instance, artemisinin might have effects on human cancers [4]. Artemisinin can potentially serve as a drug against hepatitis B virus [5].

The artemisinin biosynthesis pathway has been studied by several groups for many years. The enzymes in the pathway have almost been completely isolated [6, 7]. Through mevalonate pathway (MVA) or nonmevalonate pathway (MEP), the precursor of artemisinin biosynthesis isopentenyl diphosphate (IPP) is formed [8]. HMG-CoA Reductase Gene (HMGR) is the rate-limiting enzyme of MVA [9, 10]. IPP and dimethylallyl diphosphate (DMAPP) change into each other and form farnesyl diphosphate (FPP) by farnesyl diphosphate synthase (FPS). FPP is converted to amorpha-4,11-diene by amorpha-4,11-diene synthase (ADS), which is the first committed step in artemisinin biosynthetic pathway [11, 12]. Through cytochrome P450 enzyme CYP71AV1 (CYP), amorpha-4,11-diene is oxidized to artemisinic alcohol. Then, artemisinic alcohol is oxidized to artemisinic aldehyde [13]. Artemisinic aldehyde is reduced to its dihydro form, dihydroartemisinic aldehyde, by artemisinic aldehyde Δ11 (13) reductase (DBR2) [14]. Dihydroartemisinic aldehyde is oxidized to its dihydro acid form, dihydroartemisinic acid (DHAA), through aldehyde dehydrogenase (ALDH1) [15]. The mechanism behind DHAA converted to artemisinin is still not clear [16]. Besides, the promoters of* ADS*,* CYP*, and* DBR2* have been cloned [17–22].

Although artemisinin is widely used, the yield of artemisinin cannot meet market demand [23, 24]. Plant metabolic engineering is promising to produce artemisinin. Overexpression of one or more genes in artemisinin biosynthetic pathway increases the yield of artemisinin in transgenic* A. annua*. Overexpression of* HMGR* resulted in enhancement of the artemisinin content in transgenic* A. annua* [25, 26].* FPS* overexpressing lines exhibited 3.6-fold higher content of artemisinin than wild plants [27, 28]. Overexpression of* DBR2* significantly improved the content of artemisinin to 1.59–2.26 times compared with that in control [29]. Overexpression of* HMGR* and* ADS* led to 7.65-fold higher content of artemisinin compared with nontransgenic plants [30]. Stacked overexpression of* FPS*,* CYP*, and* CPR* resulted in 3.6-fold higher content of artemisinin compared with the controls [31]. Downregulation of enzymes competitive with artemisinin biosynthesis also led to an improved yield of artemisinin. The content of artemisinin was increased by downregulation of squalene synthase (SQS), a key enzyme of sterol pathway [32, 33]. The artemisinin content of transgenic* A. annua* by downregulation of *β*-caryophyllene synthase (CPS) exhibited an increase of 54.9% compared with the wild plants [34].

Transcription factors are also used in plant metabolic engineering. WRKY transcription factors are one of the largest families of regulatory proteins in plants [35]. WRKY transcription factors contain their DNA binding domain, WRKY domain, which binds to W box (TTGAC[C/T]) [35–37]. WRKYs have many different functions, including biotic stress, abiotic stress, and trichome development [35]. One WRKY transcription factor may regulate several different processes. For example, HvWRKY38 is involved in plant development through gibberellin signaling and abiotic stress [38–40]. LtWRKY21 can activate abscisic acid (ABA) signaling pathway [41]. Overexpression of * OsWRKY45* improved drought tolerance of transgenic* Arabidopsis* [42]. Heat and drought tolerance was enhanced in transgenic rice plants by overexpressing* OsWRKY11* [43]. Overexpression of* AtWRKY25* or* AtWRKY33* increased salt tolerance and ABA sensitivity in transgenic* Arabidopsis* [44]. Overexpression of* AtWRKY25* also exhibited enhanced heat tolerance [45]. Three WRKY transcription factors, GmWRKY13, GmWRKY21, and GmWRKY54, exhibited differential tolerance to abiotic stresses [46]. BhWRKY1 was involved in the dehydration by binding to the promoter of galactinol synthase [47]. AtWRKY63 was involved in plant responses to ABA and drought tolerance in transgenic* Arabidopsis* [48]. AtWRKY15 functioned as a negative regulator of osmotic stress responses by mitochondrial retrograde regulation [49]. Many transcription factors were found to regulate key enzymes in artemisinin biosynthetic pathway. They are also quite important in plant metabolic engineering. Both AaERF1/2 and AabZIP1 can bind with the promoter of* ADS* and* CYP* and regulate their expression [50–52]. TAR1 could interact with* ADS* and* CYP* and further regulate the biosynthesis of artemisinin [53]. Ma et al. found that AaWRKY1 could bind to the W boxes of ADSpro and activate the expression of* ADS* in transient expression systems [54].

In this study, pCAMBIA2300-AaWRKY1 fusion expression vectors were constructed under the drive of 35S promoter and transformed into* A. annua*. Transgenic plants overexpressing* AaWRKY1* were analyzed. Overexpression of* AaWRKY1*  led to great enhancement of artemisinin content.

## 2. Materials and Methods

### 2.1. Plant Materials

The seeds of low artemisinin-yielding* A. annua* were obtained from our lab. Seeds were sterilized in 75% ethanol for 2-3 min, followed by 1% sodium hypochlorite solution for 8 min, and then washed with sterilized distilled water several times. Seeds were sown on Murashige and Skoog (MS) medium under a photoperiod of 16 h light/8 h dark at 22 ± 1°C. The seedlings were transferred to the soil after 10 days in greenhouse. The young leaves of* A. annua* plants were collected for RNA extraction. The leaves of plants were collected for DNA extraction 2 months after being transferred to the soil in the growth chamber. Tobacco (*Nicotiana benthamiana*) seeds were sown on soil in pots in greenhouse. The leaves of 4-week-old tobaccos were prepared for dual-luciferase assay.

### 2.2. RNA Extraction and RT-PCR

Total RNA was extracted from the young leaves of* A. annua* plants using TRIzol Reagent Kit (Invitrogen, USA) according to the manufacturer's instructions. Concentration of the* A. annua* total RNA was measured by a NanoDrop spectrophotometer (NanoDrop, Wilmington, USA) and checked by agarose gel electrophoresis. First-strand synthesis of cDNA was carried out by M-MLV Reverse Transcriptase (Promega, USA) according to the manufacturer's instructions. RNA (500 *μ*g) was reverse transcribed with 0.5 *μ*L 50 *μ*M oligo-dT primers. The 1st-strand cDNA was used as template for quantitative real-time RT-PCR (qPCR).

Expression of* AaWRKY1* and other enzymes in* A. annua* was analyzed by qPCR using the fluorescent intercalating dye SYBR-Green (Tiangen Biotech, Beijing) in a detection system (Opticon3, MJ Research). The qPCR was performed as previously described [21, 55]. First single-stranded cDNA was used as the template in 20 *μ*L reaction mixture including 10 *μ*L SYBR Premix Ex Taq™ and 10 pmol of each primer. The primers used were listed in Table 1. The qPCR was performed first at 95°C (30 sec) with 40 cycles at 95°C (5 sec) and 56°C (30 sec) and finally a dissociation stage at 95°C (15 sec), 56°C (30 sec), and 95°C (15 sec). The data were analyzed by 2^−ΔΔCT^ method [56]. The transcript level of* A. annua ACTIN* was used as the control for qPCR analysis.

### 2.3. DNA Sequence Analysis

*AaWRKY1* was isolated by amplifying the total cDNA with the specific primers AaWRKY1-up and AaWRKY1-down. The acquired sequence was cloned to pJET1.2 vector according to the instructions (Invitrogen, USA).* AaWRKY1* sequences were analyzed using Vector NTI software (Invitrogen, USA). Bioinformatics analysis of AaWRKY1 was performed online at the NCBI database (http://www.ncbi.nlm.nih.gov/) and EBI database (http://www.ebi.ac.uk/Tools/msa/clustalw2/).

### 2.4. Dual-Luciferase Assay

The reporter and the effectors constructs were performed according to the protocols described before [52, 57]. In this study, 2.9 kb and 2 kb fragments upstream of ATG of* ADS* and* CYP* gene were cloned into pGREEN0800 LUC vector, which contain both firefly luciferase (LUC) and renilla (REN) under the 35S promoter. The construct 35S::CFP reporter was used as negative control.* Agrobacterium tumefaciens* strain GV3101 containing effector constructs (35S-CFP, 35S-AaWRKY1) or reporter constructs was adjusted to OD600 = 0.6 in MS medium with 200 *μ*M acetosyringone and 10 mM MES (pH 5.6). The mixture was incubated for 3 hours in room temperature. Mix the effector and reporter (MS medium/effector/reporter = 2 : 2 : 1, vol/vol/vol) and infiltrate the mixture into 4-week-old tobacco (*Nicotiana benthamiana*) leaves. The tobaccos were put in the dark overnight. The leaves of tobacco were collected after 2 days for dual-LUC assay according to the manufacturer (Promega, USA).

### 2.5. Construction of Overexpression Vectors and* A. annua* Transformation

The pCAMBIA2300 vector was used for the transformation of* A. annua. AaWRKY1* was amplified with specific primers AaWRKY1-up and AaWRKY1-down (Table 1) and then was cloned to the pJET1.2 vector. The pJET1.2-AaWRKY1 construct was digested with* Kpn* I and* Sac* I. The small fragment was extracted and was inserted into pCAMBIA2300 vector digested with the same restriction enzymes. The acquired construct pCAMBIA2300-AaWRKY1 was introduced into* E. coli* DH10b and then was grown in LB medium containing kanamycin. The acquired construct was introduced into* A. tumefaciens* strain EHA105 by the freeze and thaw method.

The seedlings were collected for stable* A. annua* transformation when they grew to 5 cm in length. The seedlings were cut into 0.5 cm diameter discs for the cocultivation. The plant transformation was carried out as previously described [21, 32, 58]. The* A. tumefaciens* strain harboring* AaWRKY1* was cocultivated with the leaves discs at 28°C in the dark for 2 days. The explants were washed with water several times after cocultivation. The explants were screened in selective shoot-induction medium MS1 (MS0 + 2 mg/L N^6^-benzoyladenine + 0.2 mg/L naphthalene-1-acetic acid + 50 mg/L kanamycin). The plantlets acquired were transferred into rooting medium MS2 (MS0 + 0.3 mg/L naphthalene-1-acetic acid) for root induction. When roots were formed, regenerated* A. annua* plants were transferred into soil in the growth chamber. After genomic DNAs were extracted by CTAB method [55, 59], using primers 35S and AaWRKY1-down (Table 1),* AaWRKY1* transgenic plants were identified by PCR analysis.

### 2.6. Artemisinin Quantification

The 10th leaves from apical meristem on the main stem of five* A. annua* plants were collected. The leaves were immerged in 5 mL chloroform in 15 mL tubes. The mixture was shaken for 5 min. The leaves from the tubes were collected and weighed when they dried completely in drying oven at 50°C. The extract was also dried completely in fume hood and then dissolved in 3 mL methanol, followed by ultrasonic treatment with 35 W at 40°C. The final supernatant was filtered through a 0.25 *μ*m filter.

For each sample, 20 *μ*L of the filtrate was injected into an injection port of HPLC. The samples were analyzed using a Waters Alliance 2695 HPLC system coupled with a Waters 2420 ELSD detector (Milford, USA). The filtrate was separated on a 5 *μ*m C18 column with 1 mL/min flow rate. A mixture of 60% (v/v) methanol in water was used as mobile phase. The ELSD conditions were optimized at a nebulizer gas pressure of 345 kPa and a drift tube temperature of 45°C. The content of artemisinin was indicated as mg/g dry weight (DW). Three biological repeats were measured for each sample.

## 3. Results and Discussion

### 3.1. Cloning of* AaWRKY1*

According to the previous study of* AaWRKY1* (GenBank accession number FJ390842),* AaWRKY1* gene sequence was amplified by PCR using* A. annua* cDNAs as templates, reversely transcribed from total RNAs. The sequence was acquired from low artemisinin-yielding* A. annua *var. 38 obtained from our lab, using primers AaWRKY1-up and AaWRKY1-down. Then, it was cloned into pJET1.2 vector and sequenced.

The acquired* WRKY* gene contained 936 bp open reading frame (ORF) sequence and encoded a protein of 311 amino acids. Alignment of the protein sequences of the acquired WRKY with the other two cloned WRKYs showed that they had high similarity on protein sequences. Four amino acids are different between the acquired WRKY and AaWRKY1, and five amino acids are different between the acquired WRKY and another AaWRKY1 cloned by Han et al. in 2014 [60]. The putative WRKY motif and the amino acid residues which form the zinc finger were conserved in all the three WRKYs (data not shown). Although there are some differences in these sequences, the similarity among these three genes was more than 97% and the WRKY motif and the amino acid residues which formed the zinc finger were identical. Therefore, we consider that they are alleles of AaWRKY1 from different ecotypes of* A. annua.*

### 3.2. *AaWRKY1* Was Induced by MeJA

**Figure 1:**
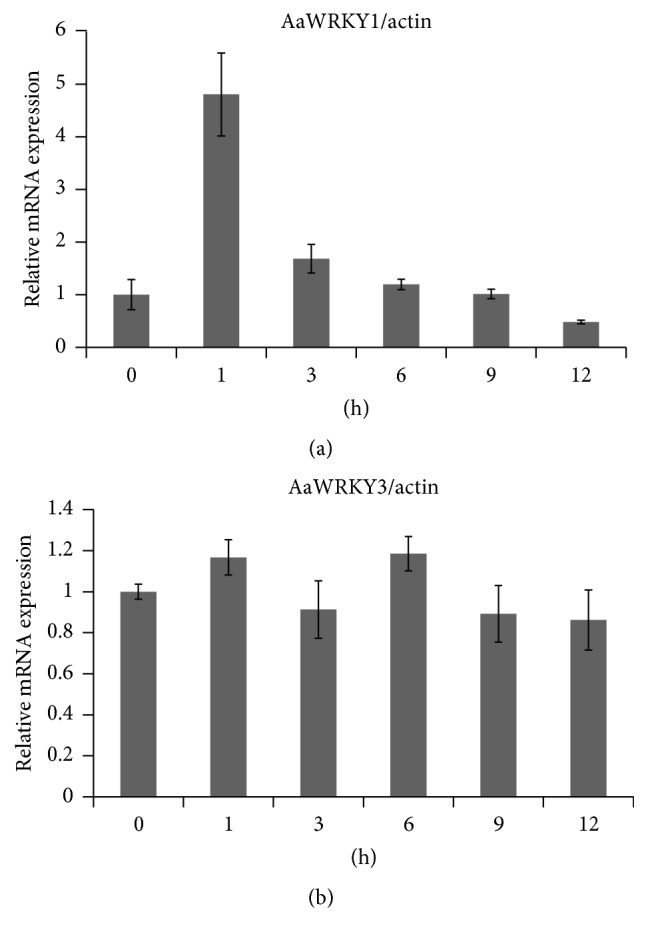
*AaWRKY1*  and* AaWRKY3* expression in response to MeJA treatment. (a)* AaWRKY1* expression in response to MeJA treatment. (b)* AaWRKY3* expression in response to MeJA treatment. Three biological repeats were measured for each sample.

Methyl jasmonate (MeJA) is an effective elicitor in artemisinin biosynthesis [61]. The expression of* ADS* and* CYP*, the two key enzymes of artemisinin biosynthetic pathway, was activated by MeJA. The expression level of* ADS* was increased rapidly and peaked in 1 h after treatment, while the expression of* CYP* was induced slowly and peaked in 9 h after treatment [51]. In this study, the expression of* AaWRKY1* and* AaWRKY3* (accession number GU299481) previously cloned in our lab was tested. The expression of* AaWRKY1* was increased rapidly and peaked in 1 h after treatment with 100 *μ*M MeJA (Figure 1(a)), while the expression of* AaWRKY3* was not changed at the same condition (Figure 1(b)). Ma et al. reported that the expression of* ADS* was rapidly induced by 0.3 mM MeJA in 0.5 h and peaked by semiquantitative RT-PCR.

JA is linked with artemisinin biosynthesis and trichome initiation and development. JA promoted the formation of both glandular and filamentous trichomes [61]. JA also increased the expression of artemisinin biosynthesis genes [61–63]. Many transcription factors induced by JA have been reported to regulate the artemisinin biosynthesis. AaERF1/2 could bind with the promoters of* ADS* and* CYP* and increased the accumulation of artemisinin in overexpression plants [51]. AaORA positively regulated the expression of* ADS*,* CYP*, and* DBR2* and significantly increased the content of artemisinin in overexpression plants [50]. These transcription factors above are strongly induced by JA rapidly. In this study, the expression of* AaWRKY1* was also increased rapidly by JA. These results suggested that AaWRKY1 might regulate artemisinin biosynthesis via MeJA.

### 3.3. *AaWRKY1* Activated the Expression of ADS and CYP in Tobacco

**Figure 2:**
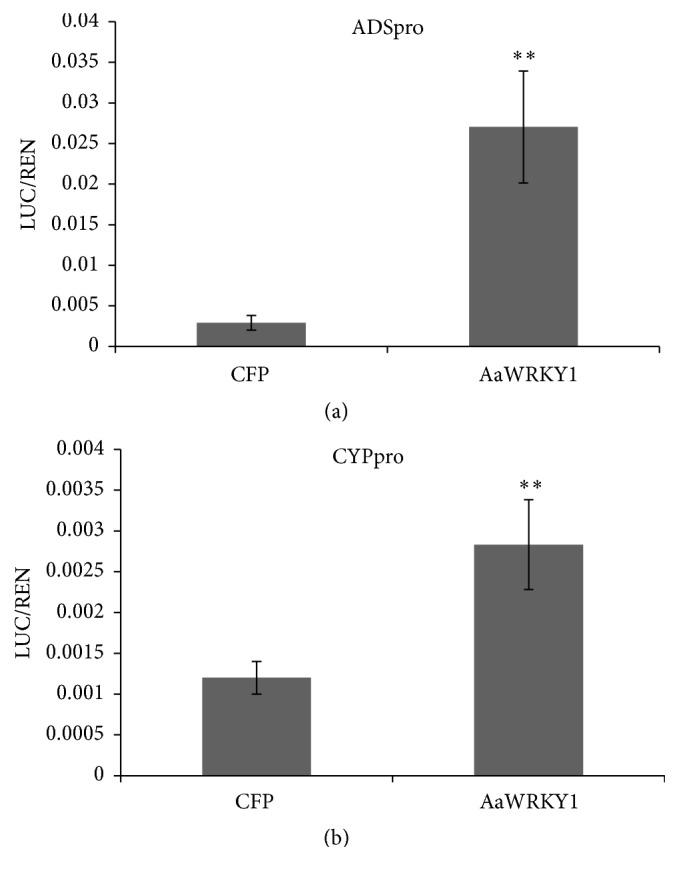
AaWRKY1 activated ADSpro and CYPpro in transient tobacco system. (a) AaWRKY1 activated ADSpro in transient tobacco system. (b) AaWRKY1 activated CYPpro in transient tobacco system. Three biological repeats were measured for each sample. Statistical significance was determined by Student's *t*-test (^*∗∗*^ *P* < 0.01).

In order to investigate the function of AaWRKY1, a dual-luciferase (dual-LUC) assay was performed in transient tobacco expression system. The promoters of* ADS* and* CYP*, named ADSpro and CYPpro, were ligated to the pGREEN0800-LUC vector as the reporters, respectively. For the negative control, a CFP protein under the 35S promoter was coinfiltrated with reporters. AaWRKY1 activated the expression of the* ADS* and* CYP* promoters by showing higher value of LUC/REN compared with the CFP control in tobacco (Figure 2). The expression of* AaWRKY1* plus ADSpro sample was increased to 9.3-fold compared with the CFP control (Figure 2(a)). The expression of* AaWRKY1* plus CYPpro sample was increased to 2.4-fold compared with the CFP control (Figure 2(b)). These results showed that AaWRKY1 activated both* ADS* and* CYP* promoters in plants.

AabZIP1 can bind with the promoters of* ADS* and* CYP* in tobacco and can improve the content of artemisinin in overexpressing transgenic plants [52]. AaWRKYs might have the same function in overexpressing transgenic plants. Ma et al. showed that AaWRKY1 binds with ADSpro and activates the expression of ADSpro in tobacco [54]. The promoter of* CYP*, the second key enzyme in artemisinin biosynthetic pathway, was cloned in 2012 [18]. Two putative W boxes were found in the promoters of* ADS* and* CYP* [17, 18]. This indicates that AaWRKY1 might bind with the promoters of* ADS* and* CYP* on W box site. In this study, AaWRKY1 activates the expression of* ADS* and* CYP* promoters* in vivo*. This suggests that AaWRKY1 might regulate the content of artemisinin in plants by regulating the expression of* ADS* and* CYP*.

### 3.4. Stable Transformation and PCR Analysis

**Figure 3:**
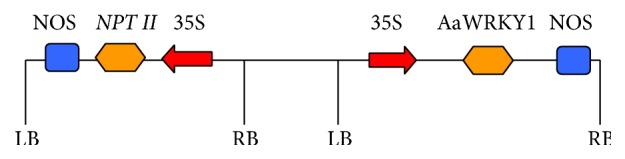
Construction of pCAMBIA2300-*AaWRKY1* vector.

**Figure 4:**
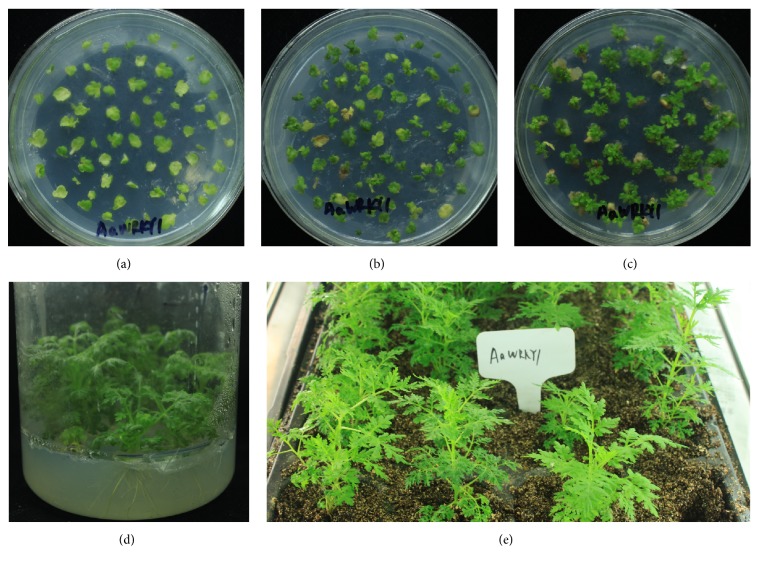
(a) Cocultivation of explants at 28°C in the dark for 48 h. (b-c) Shoot formation in shoot-induction medium. (d) Roots were formed in rooting medium. (e) The rooted plantlets were transferred into soil for further growth.

**Figure 5:**
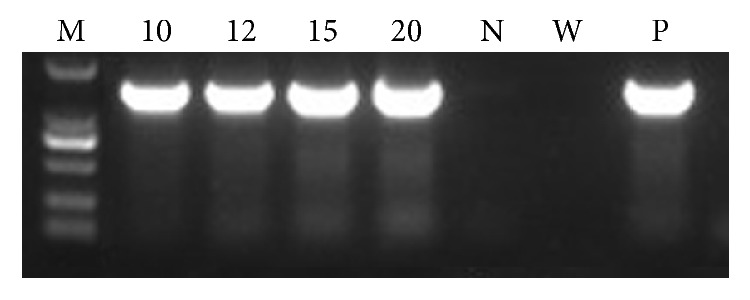
PCR analysis in transgenic plants overexpressing* AaWRKY1*. Forward primer 35S and reverse primer AaWRKY1-down were used in PCR analysis. M: DNA size marker DL2000; 10, 12, 15, and 20: independent lines of transgenic plants overexpressing* AaWRKY1*. N: nontransgenic* A. annua* control plant; W: water control; P: positive plasmid control.

**Figure 6:**
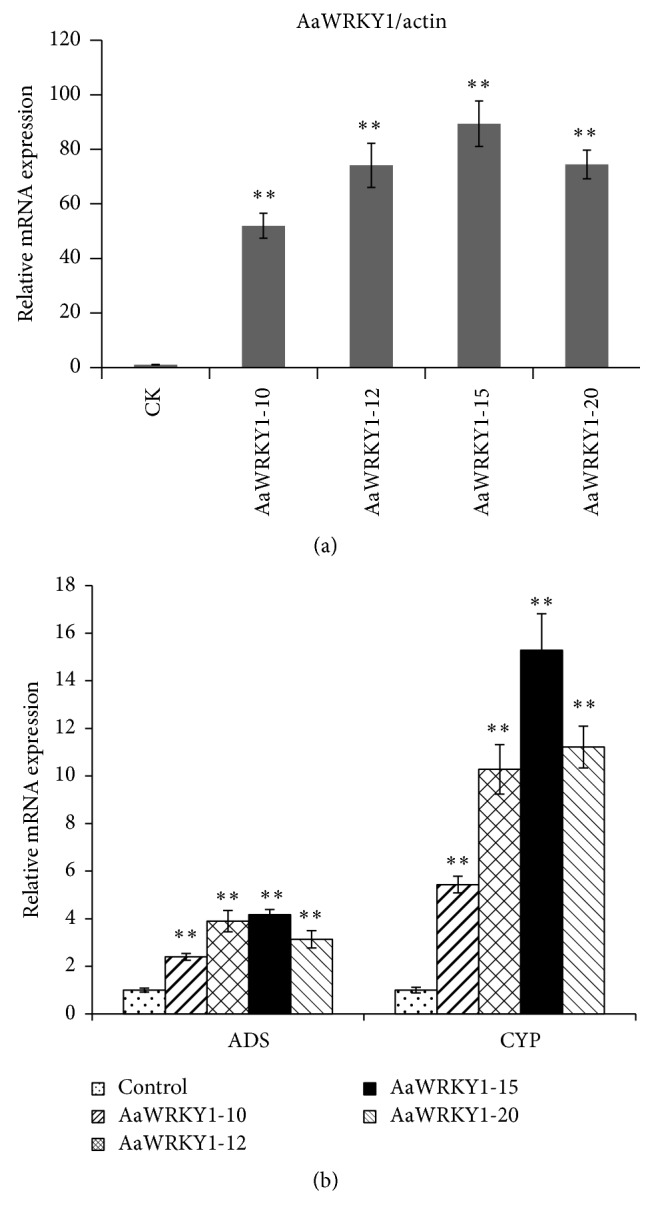
Expression analysis of* WRKY1* and enzymes in artemisinin biosynthetic pathway in transgenic plants overexpressing* AaWRKY1*. (a) Expression analysis of* WRKY1* in artemisinin biosynthetic pathway in transgenic plants overexpressing* AaWRKY1*. (b) Expression analysis of enzymes in artemisinin biosynthetic pathway in transgenic plants overexpressing* AaWRKY1*. Three biological repeats were measured for each sample. Statistical significance was determined by Student's *t*-test (^*∗∗*^ *P* < 0.01).

To investigate the function of* AaWRKY1* in artemisinin biosynthesis, pCAMBIA2300-AaWRKY1 expression vector was constructed (Figure 3). The acquired construct was stably transformed into* A. annua* by* A. tumefaciens*-mediated leaf-disc transformation method. The leaf discs were used as explants (Figure 4(a)) and were cocultivated at 28°C in the dark for 48 hours. The shoots were regenerated after changing the selective shoot-induction medium every two weeks (Figures 4(b)-4(c)). The roots were formed in rooting medium every two weeks (Figure 4(d)). Then, the rooted plantlets were transferred into soil for further growth (Figure 4(e)). Seventy-six plants acquired were analyzed. Thirty-five transgenic* A. annua* plants overexpressing* AaWRKY1* on kanamycin-selective medium were obtained and confirmed by PCR (data not shown). Four independent transgenic lines with high expression level of* AaWRKY1* were used for further analysis (Figures 5 and 6(a)).

### 3.5. Expression of Enzymes and the Content of Artemisinin in Transgenic Plants

In* AaWRKY1*-overexpressing transgenic plants, the expression of* AaWRKY1* was greatly increased by about 50- to 90-fold compared with that in the nontransgenic control plants (Figure 6(a)). There were no obvious differences in plant height and morphology between transgenic plants and wild type plants.

The expression of the key enzymes in artemisinin biosynthetic pathway was also measured. Compared with that of nontransgenic control plants, the expression of* ADS* was increased by 1.5- to 3.0-fold, while the expression of* CYP* was increased by 4.4- to 14.0-fold (Figure 6(b)).

**Figure 7:**
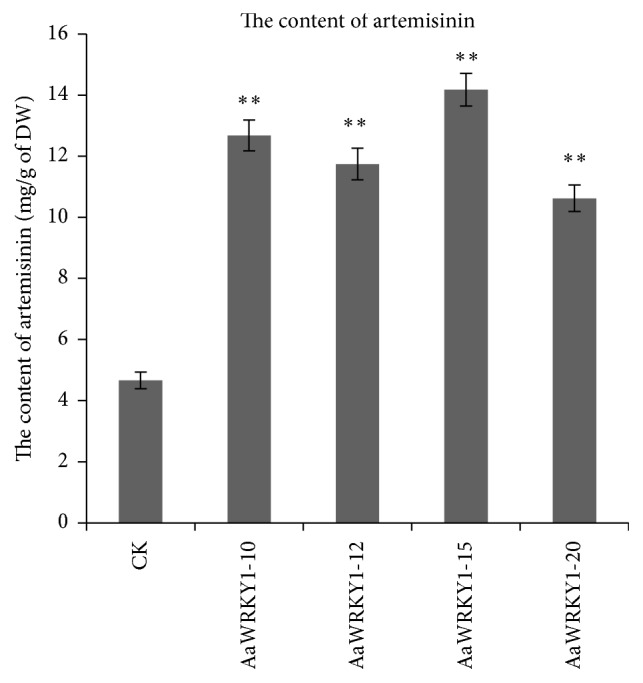
The content of artemisinin in transgenic plants overexpressing* AaWRKY1*. Three biological repeats were measured for each sample. Statistical significance was determined by Student's *t*-test (^*∗∗*^ *P* < 0.01).

Furthermore, the content of artemisinin in the transgenic plants overexpressing* AaWRKY1* was measured. Artemisinin content in transgenic plants was significantly higher than that in nontransgenic control plants. The artemisinin content was increased by 1.3- to 2.0-fold compared with nontransgenic control plants (Figure 7).

Previous studies showed that AaWRKY1 could bind to the promoter of* ADS* and activate the expression of enzymes in artemisinin biosynthetic pathway using transient expression systems [54]. Under the 35S promoter, the content of artemisinin in transgenic plants containing* AaWRKY1* was slightly increased in the background of high artemisinin-yielding* A*.* annua* var. Chongqing [60]. The content of artemisinin in transgenic plants containing* AaWRKY1* was greatly increased in the background of low artemisinin-yielding* A. annua *var. 38 in this study. At the same time, only the expression of* CYP* was increased in transgenic plants containing* AaWRKY1* in the background of high artemisinin-yielding* A*.* annua *var. Chongqing, while both expression levels of* ADS* and* CYP* were increased in transgenic plants containing* AaWRKY1* in the background of low artemisinin-yielding* A. annua *var. 38. This may explain why the artemisinin yield was dramatically increased in low artemisinin-yielding* A. annua *var. 38 in this study. It indicates that some other coactivators existed in low artemisinin-yielding* A. annua *var. 38 or corepressors existed in high artemisinin-yielding* A*.* annua *var. Chongqing to regulate the expression of key enzymes in artemisinin biosynthetic pathway together with AaWRKY1. Alternatively, the differences of AaWRKY1 or promoters between high artemisinin-yielding* A*.* annua *var. Chongqing and low artemisinin-yielding* A. annua *var. 38 could also lead to the different content in transgenic plants. Therefore, AaWRKY1 could regulate* ADS* and* CYP* promoters, so it could greatly improve the content of artemisinin in low artemisinin-yielding* A. annua *var. 38.

## 4. Conclusions

In sum, AaWRKY1 could regulate the expression of both* ADS* and* CYP* in low artemisinin-yielding* A. annua *var. 38 and greatly increase the yield of artemisinin. These results may provide some new insight into the research on plant metabolic engineering in the future.

## Figures and Tables

**Table 1:** Primers used in this study.

Primers	Primer sequences
Actin-RT-up	CCAGGCTGTTCAGTCTCTGTAT
Actin-RT-down	CGCTCGGTAAGGATCTTCATCA
ADS-RT-up	GGGAGATCAGTTTCTCATCTATGAA
ADS-RT-down	CTTTTAGTAGTTGCCGCACTTCTT
CYP-RT-up	CGAGACTTTAACTGGTGAGATTGT
CYP-RT-down	CGAAGTGACTGAAATGACTTTACT
CPR-RT-up	GCTCGGAACAGCCATCTTATTCTT
CPR-RT-down	GAAGCCTTCTGAGTCATCTTGTGT
DBR2-RT-up	GCGGTGGTTACACTAGAGAACTT
DBR2-RT-down	ATAATCAAAACTAGAGGAGTGACCC
ALDH1-RT-up	CATCGGAGTAGTTGGTCACAT
ALDH1-RT-down	GGAGTATGTTCGGCAGGCTT
AaWRKY1-up	gaaggtaccATGGAAAGTGTTTGTGTTTATGG
AaWRKY1-down	gccgagctcTTAAAATTTGAAATCAAGGTCTA
35S	TTCGTCAACATGGTGGAGCA
AaWRKY1-RT-up	GAATCCAGGTGAAATGCTCT
AaWRKY1-RT-down	GTGCCAAATGGTTCTAAAGG
